# The upper level: examining the risk of excess micronutrient intake in pregnancy from antenatal supplements

**DOI:** 10.1111/nyas.14103

**Published:** 2019-05-15

**Authors:** Alison D. Gernand

**Affiliations:** ^1^ Department of Nutritional Sciences the Pennsylvania State University University Park Pennsylvania

**Keywords:** upper tolerable intake level, excess, vitamins, minerals, prenatal, antenatal

## Abstract

Micronutrient deficiencies are prevalent and co‐occurring among pregnant women in low‐ and middle‐income countries (LMIC). To prevent and treat deficiencies, antenatal vitamin and mineral supplements are the most common interventions during gestation. With most micronutrients, there can be health risks when intake regularly exceeds a high amount, and an upper threshold value set by the United States and Canada, the World Health Organization, and other groups is commonly called an upper intake level (UL). This review summarizes what is known about risks in pregnancy when ULs are exceeded and assesses the potential risk of exceeding the UL if a pregnant woman is taking a multiple micronutrient supplement. Overall, there is limited information on pregnancy‐specific risks from excess intake. When assuming high dietary intake plus the amount in a standard multiple micronutrient supplement (with 30 mg of iron), only niacin and iron would be expected to slightly exceed the UL. Known risks for this level intake for each nutrient are transient and mild.

## Introduction

The science of nutrition includes investigating the level of nutrients and energy needed for health across age, biological sex, and physiological status. Originally, vitamins were discovered by identifying that some components of foods, only present in small amounts, could be essential for life. From beriberi to scurvy to rickets, the earliest focus on micronutrients stemmed from the morbidity and mortality that resulted from not getting enough. Thankfully, there are many mechanisms to preserve nutrient status across a wide range of intake. Compensatory actions in the human body include increased or decreased absorption, excretion, and storage—homeostatic mechanisms that are altered in pregnancy in response to the increased need for and use of micronutrients. In general, there is a higher risk of excess from minerals and fat‐soluble vitamins (more likely to be absorbed and stored) compared with water‐soluble vitamins (limited absorption and easier to excrete).

Micronutrient status is of particular importance during pregnancy when the fetus and placenta are growing, the mother is going through a wide range of physiological changes to support the pregnancy, and mammary glands are developing for lactation.^4^ The widespread prevalence of deficiencies in micronutrients^4^, ^5^ and the enormous benefits of antenatal supplementation are well known.^6^, ^7^ The United Nations International Multiple Micronutrient Antenatal Preparation (UNIMMAP) supplement has been the most commonly produced and tested in pregnancy in low‐resource settings.^8^ Although the public health burden clearly rests with deficiency, as we work to prevent deficiencies globally we must be diligent in preventing the risk of excess intake when food, fortified food, and supplements are regularly consumed. This review broadly discusses the risks of excess micronutrient intake in pregnancy and specifically focuses on the potential risks when a UNIMMAP supplement is consumed daily. The work was conducted as part of a task force organized by the New York Academy of Sciences and commissioned by the Bill & Melinda Gates Foundation.

## Essential micronutrients

Nutrients are substances found in food or water that are needed for functions of life including metabolism, growth, repair, and reproduction. Essential nutrients are those that the human body cannot produce (or cannot produce enough of) and that people must obtain externally to live. Broadly, these are grouped as carbohydrates, protein, lipids, water, vitamins, and minerals. Nonessential nutrients and other food compounds, such as phytochemicals, can have a beneficial impact on health even though there is no overt requirement. For every essential nutrient, there is a healthy range in which a person does not get too little or too much, and this range is different across age, sex, and physiological status. It is a huge challenge of nutritional science to estimate this range, and in particular, there are sparse data on pregnant women. Adverse effects of deficiency and toxicity are particularly difficult to assess for micronutrients: some are not yet known, some are hidden because assessment is impossible or too invasive, and some are not measured because of the high cost. These issues are exacerbated in pregnancy owing to concerns for the developing embryo/fetus and the inability to assess the fetus directly in utero. Answering the question of how much is also complicated by the different chemical forms of nutrients, the matrix of food, the timing and amount of individual doses, the source (e.g., food versus water), and the size and metabolism of an individual. Further, many micronutrients have overlapping functions.

As it stands, micronutrient deficiencies are common yet challenging to assess. They are called “hidden hunger” because individuals can look healthy while lacking essential vitamins and minerals. In addition, thirst and hunger mechanisms do not drive desire specifically for foods high in micronutrients. Because pregnancy is such a critical life stage, irreparable harm can occur when micronutrients are lacking. As micronutrient‐poor diets are common globally in women of reproductive age, the most common strategy for getting essential vitamins and minerals to pregnant women has been through supplementation. Less is known and documented on high intake of micronutrients during pregnancy.

## Upper intake levels in pregnancy

In an effort to assess individuals and populations, recommendations for average daily intake—both enough and too much—have been established by individual countries, groups, and the World Health Organization (WHO). Considerations around toxicity are similar for nutrients and non‐nutrient environmental exposures (e.g., carcinogens), with the key exception that essential nutrients are not only beneficial in lower amounts, but they are also indispensable. It is important to note that values are set for healthy people with good baseline micronutrient status, which is often not true for women in low‐ and middle‐income countries (LMIC). Upper intake levels (ULs) may be used to guide clinical care but can be different when health care professionals are monitoring status and side effects closely. Particularly, when a pregnant woman has a known micronutrient deficiency (commonly iron), intakes above the UL may be needed during a limited timeframe to correct the deficit.

## The U.S. and Canada upper intakes

The United States and Canada jointly developed a set of references known as the dietary reference intakes (DRIs), the model for which was established in the 1990s.^9^ These values were intended to expand the original recommended dietary allowance (RDA), and establish rigorous methods to determine values for consistency. Ahead of this model, there was only an RDA and little framework to determine how much was too much. The tolerable UL was established as the “highest level of continuing daily nutrient intake that is likely to pose no risk of adverse health effects” (Box 1).^10^ Not all nutrients have a UL—it is critical to note that lack of a UL can be due to lack of *data* on risk, not the lack of risk itself. To set the UL, the adverse effect is broadly defined and can include a mild, reversible effect or a negative impact on another nutrient. With regard to risk to a person, the risk is considered to increase as intake increases above the UL; in other words, the UL is not simply a threshold in which risk is equally high at all levels when crossed.

**Box 1. Opening paragraph for the UL section in the DRI reports^1^, ^2^, ^3^**

1 “The Tolerable Upper Intake Level (UL) is the highest level of daily nutrient intake that is likely to pose no risk of adverse health effects in almost all individuals. Although members of the general population should be advised not to exceed the UL for [micronutrient] routinely, intake above the UL may be appropriate for investigation within well‐controlled clinical trials. In light of evaluating possible benefits to health, clinical trials of doses above the UL should not be discouraged, as long as subjects participating in these trials have signed informed consent documents regarding possible toxicity, and as long as these trials employ appropriate safety monitoring of trial subjects. Also, the UL is not meant to apply to individuals who are receiving [micronutrient] under medical supervision.”

Importantly, the UL may be set based on all intake from food, water, and supplements or based on supplements and fortified food sources alone, depending on the data that established the risk. Table 1 shows the sources considered when establishing the UL. For all micronutrients with a UL, supplements are the main risk for excess consumption. For all vitamins except vitamins A and C, naturally occurring food sources do not count toward exceeding the UL. For minerals, food sources are typically considered safe but are included as part of a total intake of the nutrient. In several cases, sources were not clearly specified in Institute of Medicine (IOM) reports or data did not exist from those sources (gray shading in Table 1).

**Table 1:** Sources of micronutrients considered in establishing the tolerable upper intake levels (ULs) set by the Institute of Medicine for the United States and Canada^1^, ^2^, ^3^, ^21^

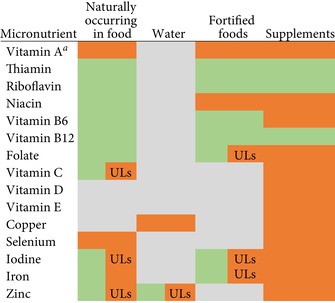

^*a*^From intake of preformed vitamin A or retinol (not to include beta‐carotene).

Note: Green shading indicates that the source is considered safe with no reports of adverse effects from intake of that source.

Gray shading indicates that the source lacks specific data related to excess, was not considered, was not specified in the Institute of Medicine (IOM) report, or does not exist in typical environments (e.g., water).

Orange shading indicates that the source was associated with adverse effects.

Green/orange (UL) shading indicates that the source was considered safe, but it is included as part of total intake for the UL.

While the RDA is based on the same mathematical model for all nutrients, the UL is based on a risk assessment framework, which does not lend itself to a single model.^10^ There are many uncertainties considered in risk assessment that fall into two main categories—data and inferences. In the case of nutrients, there can be uncertainties in data from observational studies or patient cases and there can be inferences made when studies are conducted on animal models. Overall guidelines for nutrient uncertainties have been detailed by the DRI committee,^10^ and specifics for each nutrient are within individual DRI reports. A goal of the process is to identify a no‐observed‐adverse‐effect level (NOAEL). The NOAEL should be the highest intake at which no adverse effects have been observed. However, it is not always possible to set a NOAEL directly, and instead, the lowest‐observed‐adverse‐effect level (LOAEL) is assessed and the NOAEL is estimated using an uncertainty factor (UF). Alternatively, a UF is set to estimate the UL directly from the LOAEL. UFs are higher when the uncertainty is greater. From a combination of the NOAEL, LOAEL, and UF, a UL is derived. Table 2 summarizes the considerations and values leading to the ULs for key vitamins and minerals. It is important to note that essentially all risk assessment data, with the exception of vitamin A, are based on nonpregnant human adults (or animal models) and applied to pregnancy.

**Table 2:** Factors considered in establishing the tolerable upper intake levels (ULs) set by the Institute of Medicine for the United States and Canada^1^, ^2^, ^3^, ^21^

Micronutrient	Uncertainty factor (UF)	Reason for UF	LOAEL	NOAEL
Vitamin A^a^	1.5	Interindividual variability in susceptibility; higher UF not justified owing to substantial data showing no adverse effects at doses up to 300 μg/day	ND	4500 μg/day
Thiamin	ND	ND	ND	ND
Riboflavin	ND	ND	ND	ND
Niacin	1.5	Transient nature of flushing; smaller UF not justified because it is applied to LOAEL (not NOAEL)	50 mg/day	ND
Vitamin B6	2.0	Limitations of the data involving pyridoxine doses <500 mg/day	500 mg/day	200 mg/day
Vitamin B12	ND	ND	ND	ND
Folate	5	Severity of neurological complications; use of LOAEL (rather than NOAEL)	5 mg/day	ND
Vitamin C	1.5	Mild, reversible nature of osmotic diarrhea and low uncertainty of dose range that causes it	3 g/day	Extrapolated from LOAEL (2 g/day)
Vitamin D	2.5^b^	Uncertainties of risks related to all‐cause mortality and chronic diseases	ND	10,000 IU/day
Vitamin E	36	Several sources of uncertainty were used: UF of 2 to extrapolate LOAEL to NOAEL; UF of 2 to extrapolate from subchronic to chronic intake; UF of 3 to extrapolate from experimental animals to humans; UF of 3 to account for interindividual variation in sensitivity. Final UF = 2×2 × 3×3 = 36	500 mg/kg body weight/day (∼1000 mg/day)	Extrapolated from LOAEL (250 mg/kg body weight/day)
Copper	1.0	NOAEL considered to be protective of the general population; larger UF not justified owing to a large database in humans indicating no adverse effects from daily consumption of 10−12 mg/day	ND	10 mg/day
Selenium	2	To protect sensitive individuals; the toxic effect is not severe, but may not be reversible, so UF should be >1	ND	800 μg/day
Iodine	1.5	Little uncertainty regarding the range of iodine intakes that are likely to induce elevated TSH concentration over baseline; UF does not need to be higher because of a mild, reversible nature of elevated TSH	1700 μg/day	Extrapolated from LOAEL (1000−1200 μg/day)
Iron	1.5	To account for extrapolation from LOAEL to NOAEL; higher UF not used because of the self‐limiting nature of observed GI effects	70 mg/day	Extrapolated from LOAEL (45 mg/day)
Zinc	1.5	To account for interindividual variability in sensitivity and for extrapolation from LOAEL to NOAEL; higher UF not justified because reduced copper status is rare	60 mg/day	Extrapolated from LOAEL

a Specific to women of reproductive age.

b UF calculated from NOAEL and UL but not specified in IOM report.

LOAEL, lowest‐observed‐adverse‐effect level; NOAEL, no‐observed‐adverse‐effect level; ND, not determined.

## World Health Organization upper intakes

The WHO established ULs for nutrients where sufficient information was available as the “maximum intake from food, water, and supplements that is unlikely to pose the risk of adverse health effects from excess in almost all (97.5%) apparently healthy individuals.”^11^ ULs are based on long‐term intake and although they aim to mimic a “no observed effect level” (NOEL) as used in toxicology, the data to establish cut‐points in this way for nutrients are typically not available. In a few cases, 10 times the reference nutrient intake (RNI) value is used when there is little other information on adverse effects. On the other hand, when some information is available on adverse effects, a UF is used that can be up to a 10‐fold lowering of a level at which adverse effects have been observed. Overall, ULs set by the WHO were intended for population use. While RNIs are clearly specified in the WHO report of vitamin and mineral requirements, ULs are not always delineated in the discussion of toxicity for each nutrient.^11^ Future WHO recommendations would be improved with specific UL values for each age, sex, and physiological status, along with information on the source of each nutrient that informed the UL.

## Role of upper levels in planning and assessment

Each of the U.S. and Canadian‐based DRI values has guidance on how to use it for planning or assessment for individuals or groups. For the UL, it was designed for planning and assessment for individuals, such that diets should be planned with intake at or below this level and diets are assessed as having the possibility of overconsumption if above the UL. When assessing groups, the prevalence of those at potential risk of adverse outcomes can be calculated as the percentage of a population with dietary intake of a nutrient that is above the UL. This percentage is often overlooked or not reported owing to the focus on deficiency and/or lack of information on supplement intake;^12^, ^13^ however, individual country assessments are available in some cases.^14^

Planning individual diet recommendations should aim for intake from all sources to be below the UL. For groups, the UL is most often needed in planning population‐level micronutrient interventions, which are typically focused on supplements or food fortification. Traditionally, the goal of fortification is to achieve intake between the estimated average requirement (EAR) and UL across all groups of people consuming the fortified food (including pregnant women),^15^ and software is available to aid the process of minimizing deficiency and excess specific to each setting.^16^ New approaches have been recommended, such as using a risk−benefit analysis to aid in decision making, which could be particularly helpful when the EAR and UL are not far apart or when risks of exceeding the UL are low.^17^ More data on pregnancy will be helpful to inform these population‐level efforts. Supplementation programs in LMIC are often guided by WHO recommendations, which for pregnant women currently include a recommendation for daily iron and folic acid supplements^18^ but not multiple micronutrient tablets^19^ or powders.^20^ The process of creating supplement recommendations has included risk and benefit data from randomized controlled trials of supplementation but does not follow the model for fortification that aims to get the intake from all sources between the EAR and UL.

## Risks when exceeding the upper level in pregnancy

In general, risks of excess in humans do not occur from eating food with micronutrients that are naturally present (Table 1).^1^, ^2^, ^3^^,^ 21 Primary concerns of excess come from micronutrient supplements; additional concerns can arise when food is fortified. In the case of copper, concerns of excess in the water supply also exist. Logical concerns for excess micronutrient intake during pregnancy are different from outside of pregnancy owing to potential risks to the fetus as well as increases in nutrient absorption, plasma volume, and the glomerular filtration rate of the kidneys. Yet, pregnancy‐specific risk data were not available at the time of publishing the current ULs except for vitamin A (Table 3). Therefore, current known risks of exceeding the UL in pregnancy are the same risks as in nonpregnancy plus the risk from vitamin A. However, as the risk associated with exceeding the UL is focused on chronic intake, higher concerns during pregnancy are warranted when women are taking a daily supplement across gestation that is above the UL.

**Table 3:** Micronutrients^a^ and risks from chronic excess intake^b^^,^ 1, 2, 3, 21

Nutrient	Risk(s) used to set the UL	Other risks	Pregnancy‐specific risk
Vitamin A^c^	Liver abnormalities (adults); birth defects (women of reproductive age)	Alcohol intake increases the toxicity of vitamin A, including hepatotoxicity; acute toxicity has several transient effects, including nausea, vomiting, and headache; and bulging fontanel in infants	Birth defects
Thiamin	N/A	None identified	None identified
Riboflavin	N/A	None identified	None identified
Niacin^d^	Flushing (can include burning, itching, tingling, and reddening) from nicotinic acid that results in someone reducing or stopping supplementation	Gastrointestinal effects, liver dysfunction, and glucose intolerance (from nicotinic acid)	None identified
Vitamin B6	Sensory neuropathy	None identified	None identified
Vitamin B12	None identified	None identified	None identified
Folate^e^	In B12‐deficient individuals, high folate can precipitate or exacerbate neurological damage	Masking of pernicious anemia from B12 deficiency	
Vitamin C	Osmotic diarrhea and other gastrointestinal effects	None identified	None identified (anecdotal report of fetal vitamin C dependence)
Vitamin D	Hypercalcemia and related toxicity (anorexia and weight loss can eventually lead to vascular and tissue calcification with subsequent renal and cardiovascular damage)	May increase the risk of all‐cause mortality, certain cancers, cardiovascular disease, and fractures and falls	None identified
Vitamin E^d^	Hemorrhagic effects	Supplementation may increase the risk of hemorrhagic stroke; excessively high doses can contribute to increased prooxidative damage^e^	None identified
Copper	Liver damage	Nausea and other gastrointestinal illness	None identified
Selenium	Brittleness and loss of hair/nails	Gastrointestinal disturbances, skin rash, and garlic breath odor; fatigue, irritability, and nervous system abnormalities	None identified
Iodine	Elevated thyroid‐stimulating hormone (TSH)	Thyroid dysfunction; goiter	None identified
Iron	Gastrointestinal effects (e.g., constipation, nausea, vomiting, diarrhea, and abdominal pain)	Can reduce zinc absorption if iron and zinc supplements are taken without food (and iron to zinc ratio is high)	None identified
Zinc	Reduced copper status	Suppression of immune response; decrease in high‐density lipoprotein cholesterol; acute gastrointestinal distress	None identified

a Only micronutrients in the UNIMMAP supplement included.

b Based on most recent DRI reports from the IOM, unless otherwise indicated. Risks do not include effects of acute toxicity or accidental overdose.

c Based on intake of retinol or preformed vitamin A.

d Based on supplements and fortified food intake only; no known risks from naturally occurring niacin in food.

e From WHO 2004 vitamin and mineral requirements in human nutrition.^11^

High intake of supplemental vitamin A is well known for its risk of causing birth defects if taken early in pregnancy.^1^ The minimal dose found to cause teratogenicity is 10,000 IU per day, which would only be possible if a pregnant woman was specifically taking high‐dose supplements. However, caution is also warranted for nonpregnant females taking retinoid acne medication, which can cause the same adverse effect if pregnancy occurs while being treated. For niacin, folate, vitamin B6, and vitamin E, potential adverse effects detailed in Table 3 are only due to intake from supplements and are not pregnancy specific. The known risks associated with excess folate are limited to those with vitamin B12 deficiency (detailed further below). Risks from high vitamin D also generically apply to adults and are only due to supplements. Uniquely among nutrients classified as essential, vitamin D can be produced in the skin with sun exposure. Excess amounts, however, do not occur from skin production.

For several vitamins, including thiamin, riboflavin, vitamin B12, and vitamin C, large supplemental doses are well tolerated and there is little to no documented risk in humans.^1^, ^2^, ^3^ The low risk is due to a limited capacity for gut absorption, such that high doses are simply excreted without being absorbed. There have been two anecdotal case reports of offspring vitamin C dependence when mothers took high doses late in pregnancy; however, these reports were not substantiated or corroborated by other evidence and were not considered when setting the UL.^3^

Excess intake of minerals all have associated risks, but none are pregnancy specific (Table 3). The studies on adverse effects are almost exclusively from supplementation trials, yet different from vitamins, total intake is considered for the UL. In the case of iron, nausea and vomiting is an adverse effect documented in both pregnant and nonpregnant populations (thus not included as a pregnancy‐specific risk). Overall, there is little to no risk from exceeding intake of minerals from diet alone, and no pregnancy‐specific risks are currently known.

## Micronutrient intake from diet and potential to exceed the UL

Dietary intake of micronutrients is difficult to assess in LMIC. Among the challenges, there are wide regional differences and representative samples of populations are hard to achieve. As well, many countries do not have food composition tables and inaccuracies occur from food substitutions. Two reviews that compiled dietary intake information in pregnant women globally and compared intakes with the WHO EARs found that mean/median intakes were low for iron, zinc, and folate for most populations.^12^, ^13^ On the other hand, some mean/median intakes were higher than the EAR for vitamin A, vitamin C, thiamin, riboflavin, and niacin. In assessing risks of exceeding the UL, vitamin C has a very high UL and is not likely to be exceeded. The UL for niacin is based on intake from synthetic forms, which is not likely to be the case in these settings. For thiamin and riboflavin, there is currently no UL set by the WHO or the United States and Canada; and for vitamin A, the known risks are only due to preformed retinol, which is not likely to be the major contributor.

Several recent publications have begun to assess and address the potential for excessive intake when multiple programs and interventions overlap in a community, but these are not pregnancy specific.^22^, ^23^ A study in Australia assessed the prevalence of exceeding the UL during pregnancy and found that around 20% of women exceeded the UL for iron and folate (owing to supplements).^14^ However, most studies in LMIC do not report the full range of intake including the highest intake of micronutrients. More studies collecting robust data on diet and supplements, and comparing intake to the UL, are needed.

## Potential for excess intake when taking daily UNIMMAP

The UNIMMAP supplement was initially established in 1999 by UNICEF and WHO to contain approximately one RDA/adequate intakes (AI) for each of 15 selected micronutrients.^8^ At the convened workshop to develop the supplement, toxicity and side effects from high intake were considered. Few details were provided in the report, but the expert group concluded that using the RDA was likely the amount to best balance potential benefits and harms and that trials testing the supplement should document morbidity and adverse side effects (Annex 4). The RDAs established by the United States and Canada (as of 1998) were used because they were “the most recent and best documented.”

Since the 1999 workshop, new or updated DRI reports have been published for nine of 15 micronutrients in UNIMMAP.^1^, ^3^, ^21^ These reports include ULs (or reasons for the lack of setting a UL) for each nutrient. In comparison to current recommendations, there is some variation in the UNIMMAP formulation around 100% of the RDA/AI; however, all values are substantially below ULs. As well, more than 20 randomized controlled trials have been published comparing multiple micronutrient (often, but not always similar to UNIMMAP) to iron and folic acid supplementation in LMIC.^7^, ^24^ Conducting such trials is inherently challenging, and morbidities such as nausea and vomiting were either not collected or have not yet been reported in a way that would allow collective conclusions about the side effects of supplementation.^24^

This paper aimed to reassess potential risks of excess intake in pregnant women who take the UNIMMAP supplement daily. In the absence of data on high intake in LMIC, it was assumed that women with the highest dietary intake would be consuming approximately one RNI/RDA of any individual vitamin or mineral from foods (mean usual intake per day) and taking no other supplements. This assumption was based on the distribution of requirements in healthy populations from which the RNI/RDA values are derived—such that the RNI/RDA is approximately the highest end of the distribution (+2 standard deviations). Intake distributions are typically wider than those for requirements; however, information on the full distribution of intake is difficult to obtain. This estimate of highest dietary intake (i.e., the RNI/RDA) was then added to the amount of each nutrient in UNIMMAP and compared with either the WHO (Table 4) or IOM (Table 5) UL values. Of note, there are several micronutrients for which there is no established UL set by the WHO or the IOM.

**Table 4:** Potential micronutrient intake from diet and UNIMMAP supplement compared with the upper tolerable nutrient intake level (UL) for pregnancy set by the World Health Organization for global use

Micronutrient	Pregnancy RNI	UNIMMAP	Total	UL (WHO)^a^
Vitamin A (μg/day)	800^b^^,^ c	800	1600	3000^b^
Thiamin (mg/day)	1.4	1.4	2.8	ND
Riboflavin (mg/day)	1.4	1.4	2.8	ND
Niacin (mg/day)	18	18	**36**	35
Vitamin B6 (mg/day)	1.9	1.9	3.8	100^d^
Vitamin B12 (μg/day)	2.6	2.6	5.2	ND
Folic acid (μg/day)	600	400	**1000**	1000
Vitamin C (mg/day)	55	70	125	1000
Vitamin D (μg/day)	5	5	10	50
Vitamin E (mg/day)	7.5^e^	10	17.5	1000^f^
Copper (mg/day)	ND	2	2	ND
Selenium (μg/day)	30^g^	65	95	400
Iodine (μg/day)	200^g^	150	350	ND
Iron (mg/day)	ND^h^	30	ND	ND
Zinc (mg/day)	10^g^^,^ i	15	25	4000−8000

a Based on recommendations for adults. UL values are not specified; information regarding UL values is found in the Toxicity section for each nutrient.

b Retinol equivalents (REs).

c Recommended safe intake.

d B6 as pyridoxine.

e Based on the 19‐ to 50‐year‐old age group; there are no specific recommendations for pregnancy.

f Vitamin E toxicity occurs through excessive supplementation at high doses.

g Based on third‐trimester pregnancy recommendations.

h RNI not set because requirement depends on diet and amount of stored iron.

i Based on moderate bioavailability of zinc.

ND, not determined; UNIMMAP, United Nations International Multiple Micronutrient Antenatal Preparation.

Bold values indicate that the total is greater than, or equal to, the UL.

**Table 5:** Potential micronutrient intake from diet and UNIMMAP supplement compared with the tolerable upper intake levels (ULs) for pregnancy set by the Institute of Medicine for the United States and Canada

Micronutrient	Pregnancy RDA^a^	UNIMMAP	Total	UL (IOM)^b^
Vitamin A (μg/day)	770^c^	800	1570	3000^d^
Thiamin (mg/day)	1.4	1.4	2.8	ND
Riboflavin (mg/day)	1.4	1.4	2.8	ND
Niacin (mg/day)	18	18	**36**	35^e^
Vitamin B6 (mg/day)	1.9	1.9	3.8	100^f^
Vitamin B12 (μg/day)	2.6	2.6	5.2	ND
Folic acid (μg/day)	600	400	**1000**	1000^e^
Vitamin C (mg/day)	85	70	155	2000
Vitamin D (μg/day)	15	5	20	100
Vitamin E (mg/day)	15	10	25	1000^e^
Copper (mg/day)	1	2	3	10
Selenium (μg/day)	60	65	125	400
Iodine (μg/day)	220	150	370	1100
Iron (mg/day)	27	30	**57**	45
Zinc (mg/day)	11	15	26	40

a Based on the 19‐ to 30‐year‐old age group; values for the 14‐ to 18‐year and 31‐ to 50‐year age groups were the same except for vitamin A (750 μg), vitamin C (80 mg), and zinc (12 mg) in the 14‐ to 18‐year group.

b Based on the 19‐ to 30‐year‐old age group; values for the 14‐ to 18‐year and 31‐ to 50‐year age groups were the same except for vitamin A (2800 μg), niacin (30 mg), B6 (80 mg), vitamin C (1800 mg), vitamin E (800 mg), copper (8 mg), iodine (900 μg), and zinc (34 mg) in the 14‐ to 18‐year group.

c Retinol activity equivalents (RAEs).

d Vitamin A UL for preformed vitamin A only.

e ULs for vitamin E, niacin, and folate apply to synthetic forms obtained from supplements, fortified foods, or a combination of the two.

f B6 as pyridoxine.

ND, not determined; UNIMMAP, United Nations International Multiple Micronutrient Antenatal Preparation.

Bold values indicate that the total is greater than, or equal to, the UL.

**Figure 1:**
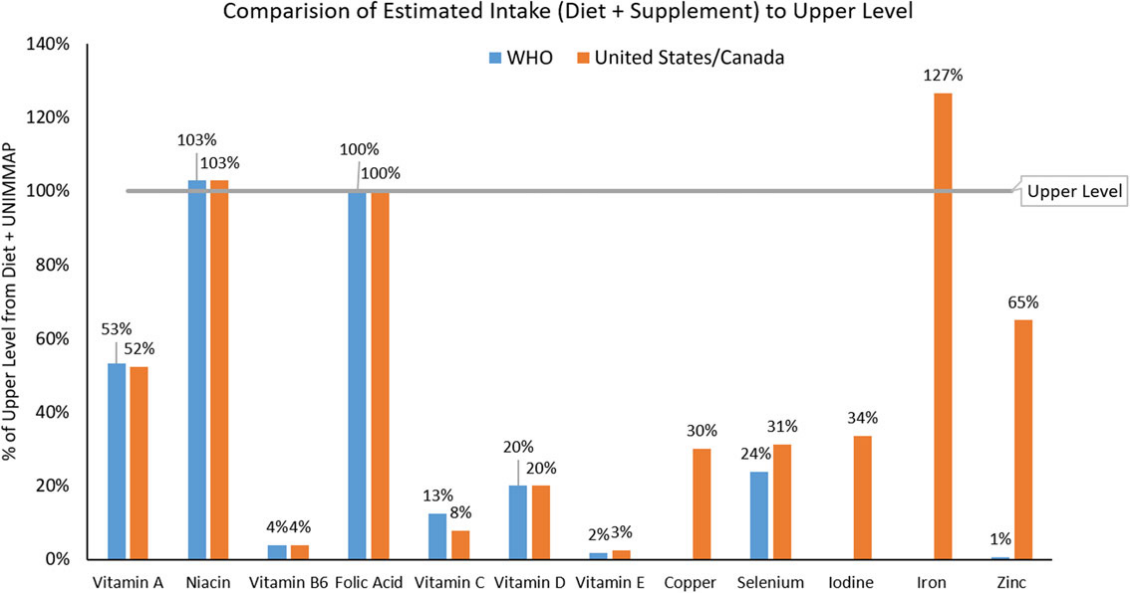
Percent of upper level hypothetically consumed if daily intake included UNIMMAP supplement plus one RDA (the United States/Canada) or RNI (WHO) from food. Note that the supplement plus the food were compared with the UL for every micronutrient for consistency, yet the UL for some nutrients does not apply to naturally occurring sources in food.

In the comparison of UNIMMAP plus dietary intake to the UL set by the WHO, only the intake of niacin exceeded the UL and folate intake was equal to the UL (Table 4). All other micronutrients were substantially below the UL as shown in Figure 1. When comparing UNIMMAP plus dietary intake to the UL set by the IOM (the United States and Canada), results were similar to those in Table 4 for 14 of 15 vitamins and minerals, including niacin and folate (Table 5). Iron intake was estimated to be higher than the IOM UL. This was not the case when comparing WHO values, because the WHO did not set an RNI or UL for iron. Other authorities have also concluded that a UL cannot be set owing to insufficient evidence.^25^ Estimating the highest intake of iron at 27 mg per day is similar to the highest observed dietary intake (without supplements) in European countries and is likely above the highest intake in LMIC. Of note, these estimates are made assuming the recommended UNIMMAP formulation with 30 mg of iron. A supplement with 60 mg of iron (the highest amount recommended by WHO) would be farther above the IOM UL but is considered appropriate in women with anemia or in settings where the prevalence of anemia in pregnancy is ≥40%.^19^

Thus, comparing a micronutrient‐rich diet plus intake of a daily UNIMMAP supplement to the UL, for both WHO and IOM values, yields amounts that are at or below the UL for all vitamins and minerals except niacin and iron. The modeled amount for folate intake was at the UL. For consistency, all values include diet and supplement compared with the UL; however, the ULs for several micronutrients, including niacin, do not include intake from naturally occurring food sources.

## Iron, niacin, and folate

Because the modeled estimates for intake of iron, niacin, and folate were at or above the UL (127%, 103%, and 100% of the UL, respectively), a detailed discussion of the potential risks for each nutrient follows. It is important to note that overall, risks of intakes closer to the UL are lower than risks of intakes far above the UL.^10^ Further, there are many uncertainties around setting the UL and data during pregnancy are particularly sparse. The UL is not meant to apply to individuals under medical care, and while this may be the case for most pregnant women in high‐income countries, women in LMIC could be part of programs to receive supplements but not have complete antenatal care.

Risks related to excess intake of iron are related to impact on the gastrointestinal tract and are more often reported when supplements are taken alone without food.^1^ A wide range of symptoms, including abdominal pain and constipation, has been reported in nonpregnant women; in pregnancy, the main documented side effects are nausea and vomiting. There is a lack of data in pregnancy for doses <100 mg/day.^1^ Overall, nausea and vomiting are among the few side effects reported in randomized trials of UNIMMAP or similar formulations.^6^, ^7^ The main concern is that women will stop taking the supplements when suffering from these ailments, although frequent vomiting is also of concern owing to nutrient loss. Iron can inhibit zinc absorption when the iron‐zinc ratio is high (25:1) but this does not occur when the ratio is lower, which is the case for UNIMMAP and most antenatal micronutrient supplements.^1^ Finally, there are unique concerns in malaria‐endemic regions. In general, there is a large body of evidence on the relationship between iron status and infections,^26^ and severe risks of iron supplementation in children in settings with malaria were brought to center stage by the Pemba trial in Tanzania.^27^ Since that time, recommendations by the WHO and others for iron supplementation include a recommendation to provide malaria prophylaxis to all pregnant women in malaria‐endemic areas.^19^, ^28^ No adverse effects were noted in antenatal multiple micronutrient supplementation trials in areas with malaria, and malaria prophylaxis was provided as part of the studies.^29^, ^30^, ^31^

For niacin, the UL is set based solely on risks due to the intake of synthetic forms from supplements and/or fortified foods. Therefore, the food intake component of the hypothetical high intake calculated in this report would not yield a higher risk. As well, the UL for niacin has been the topic of debate and some recent data suggest the value is conservative.^32^ The adverse event that sets the low UL is flushing, a mild reaction from nicotinic acid (but not nicotinamide) that can also occur after eating foods with capsaicin (e.g., spicy peppers). Flushing occurs from temporary dilation of blood vessels and can result in skin reddening, warmth, or itchiness. Serious adverse effects have not been documented until doses reach much higher amounts of 3000 mg or more per day over a long time span.^2^ The main concern with flushing is that the discomfort may cause women to discontinue taking the antenatal supplement. To our knowledge, flushing has never been reported in the context of a multiple micronutrient supplement in pregnancy.

Folate is not known to have any direct toxicity due to excess intake. The risk associated with a high intake is when an individual is also deficient in vitamin B12. Both folate and vitamin B12 deficiency cause pernicious anemia; when folate intake is high, it can correct/prevent pernicious anemia from B12 deficiency leaving it difficult to detect until more serious and threatening symptoms manifest. When women take a multiple micronutrient supplement, like UNIMMAP, that contains appropriate amounts of both folate and vitamin B12, this risk is eliminated. On the other hand, women consuming a traditional supplement with only iron and folic acid would remain at higher risk for masked B12 deficiency and potential adverse effects.

## Risk of excess from overlapping micronutrient programs

With the widespread recognition of the public health burden of micronutrient deficiencies, many programs have focused on prevention. The goal of programs is always to promote adequate, but not excessive, micronutrient intake and extensive guidance exists on the methodology to set amounts.^15^, ^17^ Yet, with numerous micronutrient‐focused programs, including fortification, biofortification, and supplementation, there is the potential for unknown overlap and resulting excess intake in certain populations. A recent review in *Annals of the New York Academy of Sciences* based on a technical consultation convened in October 2017 examined risks of excess micronutrient intake from public health interventions.^22^ At present, there is no evidence of excess intake due to overlapping micronutrient interventions; however, data are incomplete and biomarkers to indicate excess have limitations or do not exist. The authors concluded that nutrients with the highest risks from excess intake (vitamin A, calcium, copper, fluoride, iodine, iron, manganese, and zinc) should be given the closest attention and monitoring in programs.^22^ However, pregnancy was not highlighted in the report and should be the focus of program reviews in the future. Common concerns from single nutrient supplementation/fortification having negative effects on bioavailability or absorption of other nutrients are mitigated with the use of UNIMMAP or other antenatal supplements that contain multiple micronutrients.^33^

## Conclusion

The prevalence of micronutrient deficiencies in LMIC continues to overshadow any concern of excess intake, and furthermore, the potential for a “double burden” (deficiency and excess) of micronutrient malnutrition seems unlikely in pregnancy. Lack of an essential nutrient is an intractable problem in the human body, and risks of excess are often mild and short‐lived. Therefore, supplementation remains an important strategy for improving pregnancy outcomes. Importantly, there are many uncertainties in setting the RDA/RNI and UL for nutrients, particularly for pregnancy, and morbidity/side effect data from supplementation trials should urgently be published.

In this review, modeling high dietary intake coupled with a UNIMMAP supplement showed that folate intake reached the UL and iron and niacin slightly exceeded it. For folate, the only risk of excess is due to B_12_ deficiency, which is not a concern when taking a supplement that contains B_12_. For niacin, the risk of flushing is mild and only due to nicotinic acid, which is not the sole form of niacin in the diet. Finally, for iron, the risk of nausea and vomiting can be reduced or eliminated by taking the supplement with food. A supplement with 30 versus 60 mg would have a better margin of safety across populations.

It is generally under‐recognized that data on micronutrients during pregnancy are limited compared with nonpregnant adults and more information is needed on particular biological sensitivities during pregnancy. Although ULs apply to the general, healthy population, there is a high prevalence of underweight in women around the world who become pregnant and risks of excess should be given further scrutiny as more interventions are implemented. Overall, risks of exceeding the UL during pregnancy from a standard diet and micronutrient supplementation are extremely low.

## Author contributions

A.D.G. had sole responsibility for writing.

## Competing interests

The author declares no competing interests.
